# The interactomics of academia

**DOI:** 10.15252/embr.202356847

**Published:** 2023-01-26

**Authors:** Howy Jacobs

**Affiliations:** ^1^ Tampere University Tampere Finland; ^2^ La Trobe University Melbourne Vic. Australia

**Keywords:** Careers, Economics, Law & Politics

## Abstract

When scientists marry each other their chances of both finding satisfactory academic jobs diminishes. What can be done to help them fulfil their potential?

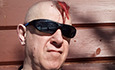

Many academics are married to other academics, which makes the job of settling into a scientific career doubly challenging. An increasing number of institutions, especially in the United States, make provision for this in their recruitment policy and procedures. But many other universities consider the offer of a tenure‐track job a singular honor for which people should be eternally grateful, instead of moaning that it will force their equally gifted spouse into unemployment. What should be done to ensure that universities treat such couples fairly, whilst maintaining their academic standards and profile?

Landing a position at a university—not just an elite college but really any—is already a demanding task. Young investigators who ask my advice about how and where to apply are invariably astonished, and assume I am exaggerating, when I tell them that they should scattershot 100 applications, expect to be shortlisted for 10 if their CV is stellar and, if lucky, get one or two offers that may or may not lead somewhere upon negotiation.

Obtaining a second position for the partner at the same university or city, even just in the same region, compounds the odds to subatomically minute levels. Unmarried candidates such as myself have an inbuilt advantage. No partner, no kids, no obligations, and free to move anywhere in the world (all offers gratefully considered). Singles are, however, out of the mainstream. Most people get married. This causes problems for relocating spouses outside of, let alone inside, academia. If one's spouse is a poet or a freelance journalist, relocation might not be a problem. But if they are in middle management in a medium‐sized company, or working as a dentist or civil servant, the options are much more limited.

When confronted with the problem, most search committee chairs, and the deans, directors, and heads of department (HoDs) who sit above them, are usually sympathetic and even prepared to take the matter higher in the organization, which candidates easily mistake as unwritten but firm commitments. However, intentions are not commitments. And a good intention that remains unfulfilled is a suppurating wound that eventually turns septic. The recruit who was brought in to great fanfare soon becomes justifiably embittered and moves on to another institution that is perceived to keep its promises more tightly. Inherent to this kind of outcome is the fact that HoDs are not themselves in post forever, so vague undertakings that they may have given become worthless when their successor takes office. The end result is career disruption for those recruited, a significant loss for the host institution, and a dent in its reputation that may impair its ability to recruit others in the future.

A common problem is that departments and faculties have few financial means to deal with the two‐body problem, as university leadership tends to reserve “strategic funding” for itself. Departments are allocated only as much funding as they “need” or have enjoyed previously, minus 5% whenever cuts are required to balance the books, which happens all too frequently. There is little room for tweaks or creative accounting, even where appropriate. HoDs can sometimes be creative, but inevitably, there are costs. I am aware of at least one case where an HoD was asked to pay out of their *own* budget the salary of an incoming academic spouse … in another faculty. I know of another case where the spouse's salary had to be taken from funding set aside for the primary recruit's startup package, handicapping both partners and the host university before they had even started. These are rare cases, though, and universities keeping the purse strings tight make such solutions increasingly difficult.

But solving this problem is not rocket science or interactomics. It simply needs some of that strategic university funding to be formally set aside to cover the costs of two‐for‐one recruitments, so that departments or institutes are not actually penalized for attracting star performers. And it also requires that rigorously fair, but flexible mechanisms are put in place to handle the practicalities of such cases.

We also need to overcome the prejudice that still exists in some quarters, that recruiting a two‐person team is somehow an abuse of the system. As if such partnerships are akin to arranged marriages designed to cheat immigration rules. Instead of regarding such couples as anomalies or cheaters to be dealt with discretely, they should be handled for what they are: another task for the recruitment office. Maybe some bold institutions might go so far as to recognize the value of recruiting two academic high‐flyers at once and actively seek out couple candidates as an opportunity rather than seeing it as a problem.

Sometimes, an academic spouse will have a track record of scholarship that merits an appointment as a tenure‐track professor in his or her own right. Others may have a different profile, for example, as a lab technician, IT expert, core facility manager, or student counselor. Placing them appropriately means a lot more than dedicated salary or startup funding. Their recruitment needs to be handled by a specialized unit, which can fairly evaluate their standing in their field and their future potential, identify how and where to fit them into the organization's existing structure and strategic plans, and negotiate with HoDs who should come to regard them as a bonus rather than a burden. This may also require cooperation between sister institutions in the same city or region, so that both partners can be placed where they truly belong, within reasonable commuting distance of their future home base, and can contribute to the pursuit of knowledge in ways that fit their goals and aptitude. Perhaps universities might even take on the burden of guiding and advising spouses who belong outside of academia, in aid of their own recruitments.

Are institutions that put effort into this process disadvantaged by it? I believe quite the opposite. Their academic staff members are more likely to stay and prosper. A feeling that the central management cares about them enhances collegiality for all. And if people are placed appropriately, in ways that engage the existing faculty, there should be no feeling that such incomers are intruders who do not really belong. Instead, such a process can prevent departments and whole universities from calcifying, and bring fresh ideas to teaching and research. Abuses of the system are rare and likely to remain so: maybe if polygamy catches on, we might have to rethink. But academia should not be a monastery. We should recognize that most people do have a partner and family, and that they are able to deliver their full potential professionally if they are able to combine love and career.

## Disclosure and competing interests statement

The author declares that they have no conflict of interest.

